# Plant Hormone Signaling Crosstalks between Biotic and Abiotic Stress Responses

**DOI:** 10.3390/ijms19103206

**Published:** 2018-10-17

**Authors:** Yee-Shan Ku, Mariz Sintaha, Ming-Yan Cheung, Hon-Ming Lam

**Affiliations:** Centre for Soybean Research of the State Key Laboratory of Agrobiotechnology and School of Life Sciences, The Chinese University of Hong Kong, Hong Kong SAR, China; ysku@ymail.com (Y.-S.K.); sintu.bmb@gmail.com (M.S.); cheungmy@cuhk.edu.hk (M.-Y.C.)

**Keywords:** biotic stress, abiotic stress, ABA, JA, SA, calcium sensors, ethylene, G-proteins, crosstalk, plant hormones

## Abstract

In the natural environment, plants are often bombarded by a combination of abiotic (such as drought, salt, heat or cold) and biotic (necrotrophic and biotrophic pathogens) stresses simultaneously. It is critical to understand how the various response pathways to these stresses interact with one another within the plants, and where the points of crosstalk occur which switch the responses from one pathway to another. Calcium sensors are often regarded as the first line of response to external stimuli to trigger downstream signaling. Abscisic acid (ABA) is a major phytohormone regulating stress responses, and it interacts with the jasmonic acid (JA) and salicylic acid (SA) signaling pathways to channel resources into mitigating the effects of abiotic stresses versus defending against pathogens. The signal transduction in these pathways are often carried out via GTP-binding proteins (G-proteins) which comprise of a large group of proteins that are varied in structures and functions. Deciphering the combined actions of these different signaling pathways in plants would greatly enhance the ability of breeders to develop food crops that can thrive in deteriorating environmental conditions under climate change, and that can maintain or even increase crop yield.

## 1. Introduction

The individual effects of isolated abiotic and biotic stresses on plants and their physiological responses to each of them have been studied extensively under experimental conditions, along with the corresponding regulatory mechanisms at the genetic and molecular levels [1,2,3,4]. However, living in the natural environment, plants are constantly challenged with a combination of abiotic and biotic stresses [5,6]. The plant responses toward the combined impact of these stresses have been largely unknown. Upon the introduction of various external stimuli, different patterns of cytosolic calcium level fluctuations are induced within seconds [7]. Therefore, calcium (Ca^2+^) signaling has been recognized as the front line of signaling events. Intracellular Ca^2+^ level is closely influenced by abscisic acid (ABA) signaling, which is an important plant hormone with multiple roles in abiotic and biotic stress responses [8]. Besides ABA, GTP-binding proteins (G-proteins) are also important components for signal transduction [9]. In this review, we will discuss the crosstalks among the signaling pathways in response to abiotic and biotic stresses. The roles of stress-responsive signaling components, including Ca^2+^ sensors, ABA-mediated signaling events, and stress-responsive G-proteins, will be discussed to spotlight the convergent points between the regulatory pathways of abiotic stress and biotic stress responses.

## 2. Ca^2+^ Sensors

The fluctuations in cytosolic calcium level upon external stimuli are known as calcium waves, which are perceived by calcium sensors. Calcium sensors are proteins that bind to Ca^2+^ and change their conformations and functions upon binding. Therefore, calcium signaling is considered to be the translator of different stimuli, and the first step in initiating downstream responses. Several calcium sensors have been shown to play roles in both abiotic and biotic stresses. Therefore, calcium sensors have also been regarded as hubs in plant responses to concurrent abiotic and biotic stresses [10].

Calcium sensors are categorized into calmodulins (CaMs) or calmodulin-like proteins (CMLs), calcineurin B-like proteins (CBLs), calcium-dependent protein kinases (CPKs), and calcium/calmodulin-dependent protein kinases (CCaMKs) [11,12].

### 2.1. CaM/CML

The CaM protein consists of two pairs of elongation factor (EF)-hands, each made up of a helix-loop-helix motif forming a Ca^2+^-binding pocket. Within each pair, the two EF-hands are connected by an α-helix. Since each EF-hand pair forms a globular structure, the whole CaM protein consists of two globular domains. Upon the binding of Ca^2+^, the conformation of CaM changes to expose hydrophobic surfaces for binding to its targets [13]. CMLs are structurally similar to CaMs, in that they are also made up of EF-hands, and they have the ability to bind to Ca^2+^. However, CMLs have low sequence similarities with CaMs. The targets of CaMs/CMLs include promoters, enzymes such as kinases and phosphatases, transcription factors, and ion channels [13,14]. The sequence divergence between CaMs and CMLs reflects their divergent properties of Ca^2+^ binding and their specific targets. Several CaMs were found to be involved in abiotic stress, while some were found to be involved in biotic stress (Table 1).

In tomato (*Solanum lycopersicum*), the expressions of *SlCaM1*, *SlCaM2*, *SlCaM3*, *SlCaM4*, *SlCaM5*, and *SlCaM6* were reported to be induced by mechanical wounding and *Botrytis cinerea* infection [15]. Transgenic tomato overexpressing *SlCaM2* had enhanced resistance to *Botrytis cinerea* infection, while transgenic tomato overexpressing the antisense *SlCaM2* and thus having reduced expression of *SlCaM2* was less resistant to the infection [15]. The expression levels of *SlCaM1*, *SlCaM2*, *SlCaM3*, *SlCaM4*, *SlCaM5*, and *SlCaM6* could also be induced by salicylic acid (SA) and jasmonic acid (JA) [15]. In *Arabidopsis thaliana*, the mutation of *AtCaM3* led to a reduced tolerance to heat stress [16]. In soybean, it was found that the expression levels of *SCaM-4* and *SCaM-5* in soybean cell suspensions were induced by *Fuasrium solani* infection [17]. The constitutive expression of *SCaM-4* or *SCaM-5* in tobacco led to enhanced resistance to infection by *Phytophthora parasitica* var. *nicotianae* [17].

For CMLs, in tomato, the expression of *ShCML44* could be induced by cold, drought, mannitol, (osmotic stress), salt, ABA, methyl-jasmonate (MeJA), and ethephon (a plant growth regulator) [18]. In *Arabidopsis thaliana*, several CMLs were found to be involved in both abiotic and biotic stresses. The mutation of *AtCML9* led to increased sensitivity to ABA, and enhanced tolerance to salt stress and water deficit [19]. On the other hand, the mutation of *AtCML9* led to reduced resistance to Pseudomonas syringae pv. *tomato* (*Pto*) strain DC3000 infection [20]. The mutation of *AtCML37* resulted in increased susceptibility to drought stress [21] and herbivory by down-regulating the JA pathway [22]. On the other hand, the mutation of *AtCML42* led to enhanced resistance to herbivory but reduced tolerance of UV-B [23]. It was suggested that AtCML42 downregulates JA sensitivity [23]. Similarly, AtCML37 and AtCML42 were also shown to be regulators of the JA response pathway [21,22,23].

### 2.2. CBL

All CBL proteins have a conserved four-EF-hand calcium-binding domain, and they specifically interact with a family of protein kinases, namely, CBL-interacting protein kinases (CIPKs) [24]. In rapeseed (*Brassica napus*), the expression of *BnCBL1* was induced by both salt and osmotic stress [25]. The overexpression of *BnCBL1* in Arabidopsis conferred improved tolerance of low inorganic phosphate stress and salt stress [25]. In *Arabidopsis thaliana*, the overexpression of *AtCBL5* resulted in improved tolerance of salt and drought stress [26]. The mutation of *CBL10* in *Arabidopsis thaliana* led to a reduced tolerance of salt stress [27]. On the other hand, in tomato, the silencing of *CBL10* led to improved resistance to *Pto* DC3000 infection [28].

### 2.3. CPK

The typical CPK protein consists of four functional domains including an N-terminal variable domain (N-VD), a protein kinase (PK) domain, an autoinhibitory junction (AJ), and a calcium-binding domain (CBD) made up of one to five EF-hands [29]. Upon binding to Ca^2+^, CPKs can then phosphorylate their specific targets [30]. The targets of CPKs include ion channels, ABA-responsive element binding factors (ABFs), and calcium ATPases (ACAs) [31]. In *Arabidopsis thaliana*, the mutation of *CPK10* led to an enhanced tolerance of drought [32]. The expression of *AtCPK3* in *Arabidopsis thaliana* protoplasts was induced by cold, salt, heat, hydrogen peroxide (H_2_O_2_), flagellin, and laminarin [33]. The mutation of *AtCPK3* led to reduced tolerance of sodium chloride (NaCl) [33]. The overexpression of *AtCPK6* led to improved tolerance of salt and drought stress [34]. It was also shown that *AtCPK3* functioned together with *AtCPK6* to regulate stomatal aperture when stimulated by ABA [35]. In maize, the overexpression of *ZmCPK4* conferred tolerance to drought stress by increasing the sensitivity to ABA [36]. In rice, the expression of *OsCPK9* was induced by ABA, NaCl, and polyethylene glycol (PEG) (osmotic stress). The overexpression of *OsCPK9* conferred tolerance to drought stress [37]. *OsCPK12* is involved in both abiotic and biotic stress. The overexpression of *OsCPK12* promoted tolerance of salt stress, increased sensitivity to ABA and increased susceptibility to blast fungus (*Magnaporthe grisea*) by reducing the accumulation of reactive oxygen species (ROS) [38].

### 2.4. CCaMK

CCaMKs have a serine-threonine kinase domain, an autoinhibitory domain, a CaM-binding domain, and a neutral visinin-like Ca^2+^-binding domain consisting of three EF-hands. The binding of Ca^2+^ leads to the autophosphorylation of the protein and enhances its affinity to CaM, resulting in the activation of kinase activity [39]. Several transcription factors, such as CYCLOPS and ZmNAC84, were reported to be the targets of CCaMKs [40,41,42]. CYCLOPS, which is a transcriptional activator regulating symbiotic nodule development, was reported to be a phosphorylation target of CCaMK [42]. On the other hand, ZmCCaMK phosphorylates ZmNAC84 and mediates antioxidant defense [41]. In soybean, the expression of *GsCBRLK* was induced by cold, ABA, NaCl, and PEG [29]. The overexpression of *GsCBRLK* in Arabidopsis thaliana led to improved tolerance of NaCl stress and reduced sensitivity to ABA [29]. In tomato, the expression of SlCCaMK in the leaf was induced by *Sclerotinia sclerotiorum* infection but repressed by *Pto* DC3000/*Xanthomonas oryzae* pv. *oryzae* (*Xoo*) infection [29]. Knocking down *SlCCaMK* reduced resistance to *S. scelrotiorum* and *Pto* DC3000 in tomato [29]. In wheat, it was found that the expression of *TaCCaMK* was reduced by ABA, NaCl, and PEG [43]. The overexpression of *TaCCaMK* in Arabidopsis led to decreased sensitivity to ABA and improved tolerance of NaCl during seed germination, but increased sensitivity to NaCl, with heavier chlorosis in seedlings [43].

### 2.5. Calcium Sensors Involved in both Abiotic and Biotic Stresses

A lot of calcium sensors were found to be involved in stress responses in plants. Among the characterized calcium sensors, some of them were found to be involved in both abiotic and biotic stresses. Examples are AtCML37, AtCML42, AtCML9, and OsCPK12.

AtCML37 and AtCML42 are regulators of JA signaling. JA has been known for its dual role in both abiotic and biotic stress signaling, and the application of JA to plants protects them against both types of stresses [44]. JA has been known to play a protective role against cold and freezing, salinity, drought, and heat [45]. Upon infection by necrotrophic pathogens, attack by insects, and wounding events, JA activates plant defense responses by activating protective genes [46]. Therefore, calcium sensors that regulate the JA pathway are probable hubs of abiotic and biotic stress responses. In addition, among the stress-responsive calcium sensors, several are related to ABA sensitivity (Table 1). Like *AtCML37* and *AtCML42*, *AtCML9* is also involved in both abiotic and biotic stresses. It was reported that *AtCML9* regulates ABA sensitivity [19]. Besides, *OsCPK12* was also reported to play a dual role in abiotic and biotic stresses [38]. It is a positive regulator of salt stress by reducing ROS accumulation, but a negative regulator of defense responses against blast fungus as a result of its ROS-reducing activity which prevents the activation of defense-related genes. It was also proposed that the dual role of OsCPK12 was due to its effect on ABA signaling, which is a major event in both abiotic and biotic stresses. ABA and JA pathways are closely interconnected. The relation between ABA and JA signaling will be discussed in more detail in Section 3. The links among the calcium sensors, ABA and JA, strongly suggest the important roles played by calcium sensors in abiotic and biotic stress responses. Table 1 shows a list of stress-responsive calcium sensors found in plants.

## 3. ABA-Mediated Stress Responses

ABA is an important phytohormone that plays multiple roles in abiotic and biotic stresses. ABA closely interacts with several other stress-response hormones including JA, ethylene, and SA. In this section, the interactions between ABA and other stress hormones will be discussed.

### 3.1. The Interactions between ABA and JA Pathways in Response to Biotic and Abiotic Stresses

Upon contact with *Pseudomonas syringae* on the leaf, flg22, a 22-amino acid epitope on the flagellum of the bacterial cell and a typical pathogen-associated molecular pattern (PAMP), is recognized by the receptor, FLS2, on the leaf epidermal cells. The receptors then trigger SA- & ABA-mediated responses to close the stomata to prevent the pathogen from gaining entry into the plant [47]. A virulent strain of *Pseudomonas syringae* then injects the phytotoxin, coronatine (COR), which mimics the action of jasmonic acid–isoleucine (JA–ILeu), which is the active form of JA, into the host plant, in order to stimulate the re-opening of the stomata. COR, thus termed an effector, functions by stimulating the biosynthesis of JA via *Jai1*, which in turn induces JA–ILeu-inducible wound-responsive genes via the transcription factor, MYC2, while repressing *PATHOGENESIS-RELATED* (*PR*) genes [48,49,50]. Thus, the wounding response becomes activated at the expense of defense response.

In the absence of the virulence factor COR or actual wounding, induction of wounding-responsive genes is inhibited by the repression of MYC2 by jasmonate ZIM-domain (JAZ) proteins [51]. However, in the presence of COR or JA–ILeu, the F-Box protein, COI1, a component of the Skp1–Cul1–F-box protein (SCF) of the ubiquitin E3 ligase complex, recruits the JAZ protein to tag it for 26S proteasome-mediated degradation [52,53]. Thus the repression on JA-inducible wounding-responsive genes by JAZ protein is lifted by JA–ILeu or COR [54].

A small GTPase protein, NOG1-2, stimulates stomatal closure during abiotic stresses, and the *nog1-2* mutant of *Nicotiana benthamiana* is more susceptible to the pathogen *Pseudomonas syringae*, due to its inability to close its stomata [55], proving its role in the re-closing of stomata opened by *Pseudomonas syringae*. NOG1-2-mediated stomatal closure is stimulated in the presence of COR, indicating that its function is dependent on JA–Ileu [48]. In Arabidopsis, JAZ9 is the protein that interacts with MYC2 and COI1 through its JA-associated (Jas) domain [56]. JAZ 9 also interacts with NOG1-2 through this same domain, and by binding to JAZ9, NOG1-2 interrupts the interaction between JAZ9 and COI1 and prevents JAZ9 from being targeted for degradation. Thus, JAZ9 can perform its role in inhibiting wounding response (i.e., stomatal re-opening) upon bacterial infection as part of the pathway in effector-triggered immunity (ETI) [48].

The commonly downregulated genes in *nog1-2* and *jaz* mutants are often induced in response to ABA or drought, indicating the cooperative nature of these two proteins in inducing ABA-responsive genes [48]. Also, the *nog1-2* mutant of *Nicotiana benthamiana* has reduced accumulation of ROS (necessary for ABA-mediated stomatal closure) in guard cells, even after external application of ABA, indicating the role of NOG1-2 in ABA-mediated stomatal closure [48]. Thus NOG1-2 acts as a point of crosstalk between the ABA and JA signaling pathways, since NOG1-2 is responsible for inducing stomatal closure as part of the ABA-dependent pathway during abiotic stress, while it causes the closing of stomata through the JA-Ileu-mediated pathway during pathogen infection [48]. Though the effect of both ABA and JA pathways is the same in terms of resulting in stomatal closure, the effects of these two hormones are opposite in the case of evoking wounding response.

**Figure 1:**
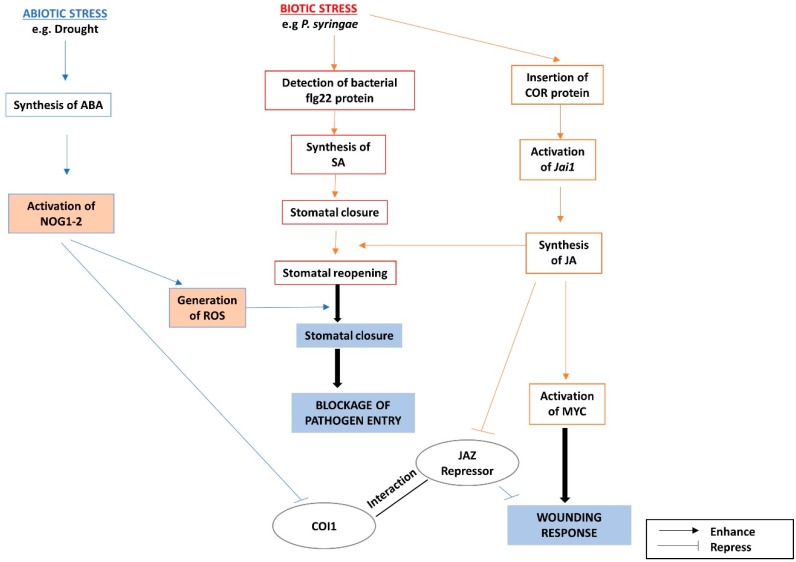
The interactions between the abscisic acid (ABA) and jasmonic acid (JA) signaling pathways under biotic and abiotic stresses are mediated through NOG1-2.

The *PDF1.2* defensin gene is induced upon fungal infection or MeJA challenge, but not by wounding or salicylic acid (SA) challenge, indicating that its induction is triggered only by pathogen attack but not by wounding [57]. *PDF1.2* requires both JA and ethylene (ET) for its activation [57], or at least the overexpression of a downstream component of the ethylene signaling pathway, such as ERF-1 [58]. Constitutive expression of *PDF1.2* occurs when *ERF-1* is overexpressed both in the ethylene insensitive (*ein2*) and JA insensitive (*coi1*) mutants [58]. At the same time, its expression is upregulated by NOG1-2 in the presence of ABA [48]. This further shows the important position of NOG1-2 as a hub between the JA/ET and ABA signaling pathways. A summary of the interactions between the ABA and JA pathways is presented in Figure 1.

### 3.2. The Interactions between ABA and Ethylene Pathways under Biotic and Abiotic Stresses

The plant ethylene receptor family consists of five members that function in conjunction with its negative regulator, CONSTITUTIVE TRIPLE RESPONSE1 (CTR1) [59,60]. CTR1 is a Raf-like protein that is associated with ethylene receptors in the endoplasmic membrane of Arabidopsis [61], and it is insensitive to ABA [60]. In the absence of ethylene, CTR1 phosphorylates and thus deactivates ETHYLENE-INSENSITIVE PROTEIN 2 (EIN2), and thus all the downstream signaling in the ethylene pathway [62]. The presence of ethylene results in the increase in the level of EIN2 by the deactivation of ETHYLENE INSENSITIVE3-BINDING F-BOX PROTEIN 1 and 2 (EBF1 and EBF2), which are responsible for the 26S proteosomal degradation of EIN2 [62,63]. Also, ethylene causes the deactivation of CTR1 [59], and therefore prevents the phosphorylation of EIN2. The C-terminal end of EIN2 is then cleaved and localized to the nucleus to activate the next component along the ethylene pathway, EIN3 [62]. The immediate target of EIN3 and the final component of the ethylene-initiated pathway in Arabidopsis is ETHYLENE RESPONSE FACTOR1 (ERF1), the constitutive expression of which elicits ethylene responses at both the transcriptional and the phenotypic levels [64]. ERF1 belongs to the Ethylene Response Factor (ERF) family of transcription factors, containing a single DNA-binding domain. Members of the ERF subfamily of the ERF family recognize the AGCCGCC motif (the GCC box) in the promoters of ethylene-inducible defense genes [65,66,67,68,69], while members of the DREB subfamily binds to the A/GCCGAC motif in the promoters of *DEHYDRATION RESPONSIVE ELEMENT* (*DRE*) genes in response to drought stress [65,70]. Among the members of the ERF subfamily in Arabidopsis, ERF1, 2 and 5 are activators, while ERF3 and 4 are repressors of gene transcription [68].

The biotic stress-inducible Arabidopsis ERF proteins are also found to be associated with various abiotic stresses such as salt, drought, cold, heat, and light [71,72,73]. AtERF6 induces many ROS-inducible genes, and thus ensures that the plant is protected against both biotic and abiotic stresses [74]. The ERF homologues in other plants are also found to be induced by biotic stresses or ethylene, and they can bind to the GCC box or DRE sequence in response to biotic and/or abiotic stresses.

Overexpression of ERF related genes such as, *OsEREBP1* in rice and *Tsi1* in tobacco, and ectopic expression of *ERF* genes such as *JERF3* from tomato and *GmERF3* from soybean in tobacco, induce the expression of *PATHOGENESIS-RELATED* (*PR*) genes and enhance the resistance to various biotic and abiotic stresses [75,76,77,78]. Ectopic expression of *GmERF3* (from soybean) and *JERF1* (from tomato) in tobacco, and overexpression of *SlERF5* (in tomato), *TaERF3* (in wheat), and *OsEREBP1* (in rice), conferred resistance to drought, possibly by binding both the *DRE* promoter sequence along with the GCC box, which results in the upregulation of ABA biosynthesis genes and the accumulation of proline [71,75,78,79,80,81]. The rice homologue (*OsWR1*) of the Arabidopsis wax/cutin-synthesizing gene, *WIN1*/*SHN1*, is also an *ERF* gene. Overexpression of *OsWR1* improves drought tolerance by inducing wax biosynthesis [82]. In Arabidopsis, *ERF1*-overexpression increases resistance to drought stress by upregulating the drought response-specific genes, *GEA6* and *LEA4-5*, accumulating ABA and proline, and closing the stomata, whereby ERF1 binds only to the *DRE* promoter sequence, whereas ERF1 binds the GCC box exclusively during biotic stress responses [71]. AtERF4, which is known to be a transcriptional repressor in response to ethylene, causes the downregulation of ABA-responsive genes when constitutively expressed [83].

*ERF1*-overexpression also causes upregulation of the heat-inducible genes, *HSP101*, *HSP70*, and *HSP23.6*, and the transcription factor, *AtHsfA3*, responsible for inducing heat-responsive genes, possibly by binding to the DRE promoter sequence [71].

Though the overexpression of many ERF transcription factors, such as those found in soybean, tomato, wheat, and tobacco, has been shown to confer salt tolerance [76,77,78,79,80,81,84], most of the research is focused on the salt tolerance mechanism in Arabidopsis. In Arabidopsis, the ethylene signaling pathway component, EIN3, activates *ETHYLENE AND SALT INDUCIBLE1* (*ESE1*), one of the salt- and ethylene-inducible genes of the *ERF* family in Arabidopsis. ESE1 confers salt tolerance by positively modulating the salt tolerance genes, *COR15A* and *RD29A* [85]. In a separate study, overexpression of *AtERF1* caused the induction of salt-responsive genes, *P5CS1*, *SRO5*, *GLP9*, and *ATOSM34*, during salt stress by binding to the DRE sequence of the promoter [71].

ERFs from various species, including Arabidopsis, tomato, and wheat [81,86,87] are also found to confer cold tolerance, possibly by upregulating the *COR* gene in an ABA-independent pathway [88].

**Figure 2:**
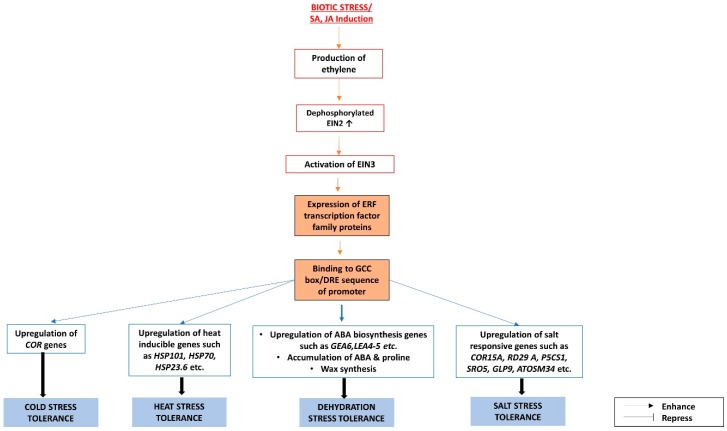
The interactions between abscisic acid (ABA) and ethylene signaling pathways under biotic and abiotic stresses are mediated through Ethylene Response Factors (ERFs).

Many ERFs that confer abiotic stress tolerance are induced not only by ethylene, but also by the other phytohormones involved in biotic stress response pathways, such as SA and JA, and hence these could be the facilitators of crosstalks between biotic and abiotic stress response pathways [76,77,78,84,89]. Characterizations of these ERFs are summarized in Table 2, and the roles of ERFs in the response pathways to various biotic and abiotic stresses are charted in Figure 2.

#### The Antagonistic Relationship between ABA and Ethylene Pathways

A unique ERF (ThERF1) from *Tamarix hispida* was found to negatively regulate drought tolerance by inhibiting superoxide dismutase (SOD) and peroxidase (POD) activities in transgenic Arabidopsis, which are both downstream components of the ABA-mediated stress response pathway [91]. It also negatively regulates salt tolerance by binding to the TTG motif of the promoter of salt-responsive genes with more affinity than to the GCC motif [94].

On the other hand, the ABA-INSENSITIVE4 (ABI4) protein binds to the promoters of the ethylene biosynthesis genes, *ACS4* and *ACS8*, in Arabidopsis, and represses ethylene production in the presence of ABA [95]. One mutated allele of the ethylene signaling pathway component, *ein2*, caused higher ABA accumulation during germination, thus demonstrating the antagonistic relationship between ABA and ethylene [96].

### 3.3. The Interactions between ABA and SA under Biotic and Abiotic stresses

In response to drought stress, ABA promotes cuticular wax biosynthesis mediated by the transcription factor, MYB96, possibly by upregulating the enzyme, 3-ketoacyl-CoA synthase 2 (KCS2), involved in very-long-chain fatty acid biosynthesis in Arabidopsis [97]. MYB96 also increases drought tolerance by upregulating the *RD22* gene, which decreases lateral root formation [98]. This phenomenon is accelerated in the super-active *myb96-1d* mutant, but it is reduced in the less-active *myb96-1* mutant [98]. MYB96 also stimulates SA biosynthesis. *SA-INDUCTION DEFICIENT 2* (*SID2*), the gene responsible for expressing isochorismate synthase, a key enzyme in the biosynthesis of SA and normally induced upon infection [99], is found to be upregulated in the super-active *myb96-1d* mutant. The *myb96-1d* mutant is more responsive to ABA-mediated signaling, and it also produces higher levels of SA and is more resistant to pathogens, compared to the wild type [100]. These mutant plants have impaired growth similar to those plants with higher levels of ABA. The dwarfism observed in these plants due to the increased ABA-mediated response can be negated by crossing with *NahG* plants [100], in which *NahG* encodes SA hydroxylase, which degrades SA [101]. This shows that the dwarfism resulting from response to ABA is actually mediated by SA. On the other hand, the stomatal closure in abiotic and biotic stresses is mediated by ABA and SA, respectively [102,103,104,105].

The ABA3 protein promotes ABA biosynthesis by activating aldehyde oxidase through sulfuration. The enzyme then catalyzes the final step of ABA biosynthesis by converting abscisic aldehyde to ABA [106]. ABA3 is also shown to induce *SID2*, as indicated by the absence of *SID2* induction upon abiotic stress in the *aba3* mutant [100].

**Figure 3:**
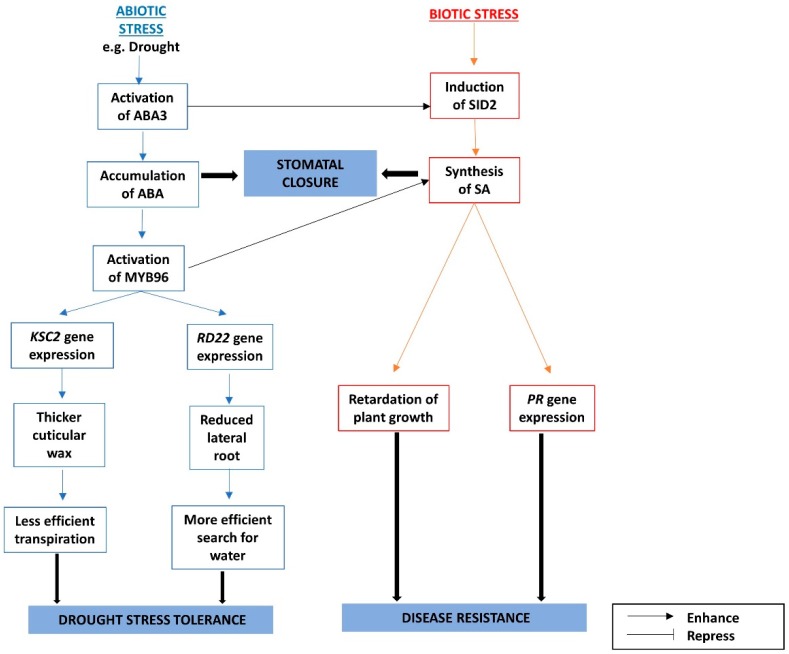
The interactions between abscisic acid (ABA) and salicylic acid (SA) signaling pathways under biotic and abiotic stresses are mediated through MYB96.

SUMO (small ubiquitin-like modifier) E3 Ligase 1 (SIZ1) negatively regulates the ABA-responsive gene, *ABI3*, since the *siz1* mutant accumulates a higher level of the ABI3 protein [107]. At the same time, it also negatively regulates SA levels, since the *siz1* mutant has a higher accumulation of SA, higher expression levels of *PR* genes, and it exhibits greater resistance to bacterial infections and drought, and these effects can be reversed by introducing the *NahG* gene [108,109]. The interactions between the ABA and SA pathways are summarized in Figure 3.

#### The Crosstalks among ABA, SA, and Phospholipids under Biotic and Abiotic Stresses

Phospholipid pathways are divided into those that activate phospholipase C (PLC), and those that activate phospholipase D (PLD). Here we will focus on the PLD pathway, which is known to be involved in both abiotic and biotic stresses. There are different genes in the *PLD* gene family, such as *PLDα*, *β*, *γ*, *δ*, *ε*, and *ζ*, classified into subfamilies [110]. PLDs mediate phosphatidic acid (PA) production, which is a typical response to viral infection, e.g., in tobacco, likely via PLDα and β, since their presence is increased in the leaf upon infection [110,111,112]. PA facilitates viral replication, possibly by aiding in the formation of an active replicase complex [112]. PLDδ was found to provide a cell wall-based defense in Arabidopsis, together with PA, in the case of fungal [112,113] or bacterial [114] infection. PLDs are also reported to participate in abiotic stress responses. For example, PLD α1, α3, and δ cause stomatal closure by inducing the synthesis of PA and the activation of Mitogen-activated Protein Kinase 6 (MPK6), thus increasing tolerance to salt stress [115,116]. Salt increases the PA content in Arabidopsis, which was found to bind to MPK6 and increase its activity. The activation of MPK6 by PA is lost in the *pldα1* mutant. MPK6 in turn activates a Na^+^/H^+^ antiporter via phosphorylation to confer salt tolerance [117]. PLDδ activity and the concentration of its catalytic product, phosphatidyl alcohols, increased upon dehydration stress; this was absent in Arabidopsis *pldδ* mutants [118]. *PLDδ* knockout mutants were found to be more sensitive to freezing stress, having less PA content, while overexpression increased the PA content, as well as conferring resistance [119]. 

Disassembly of microtubules is responsible for stomatal closure, which is inhibited in mutants that overexpress microtubules [120]. ABA, the phytohormone in the first line of response to drought and salt stress, activates PLD, which hydrolyzes membrane lipid to phosphatidyl choline (PC) [121], which is then hydrolyzed by PLDα1 to PA and choline [122]. PLDα1 and PA together depolymerize microtubules. This process is evidenced by the inability of ABA treatment to induce stomatal closure in *pldα1* mutants, but co-treatment with ABA and microtubule-disassembling drugs (orzyline and propyzamide) results in stomatal closure in these mutants [123]. PLDα1-derived PA decreases the phosphatase activity of ABI1, one of the negative regulators of the ABA signaling pathway [124].

The intracellular increase in Ca^2+^ mediated by ABA [125] helps to activate PLDs by facilitating the binding of Ca^2+^ to the catalytic domain of PLDs [126]. The downstream production of ROS by activated PLDs further increases the intracellular Ca^2+^ concentration [121]. Ca^2+^ does not play any role downstream of PLDα1, as demonstrated by the fact that exogenous Ca^2+^ treatment could dis-assemble microtubules in guard cells, and this resulted in stomatal closure in wild-type Arabidopsis but not in the *pldα1* mutant [123]. Treatment with exogenous ABA increases Ca^2+^ levels quickly in the wild type but slowly in the *pldα1* mutant, demonstrating the role of PA produced by intact PLDα1. Treatment with PA increases Ca^2+^ concentrations more in the wild type than in the *pldα1* mutant, showing the possibility that Ca^2+^ influx may occur both upstream and downstream of PLDα1 [123].. There may be a positive feedback loop, where Ca^2+^ produced by PA in turn induces PLDα1 to produce more PA. Exogenous treatment with Ca^2+^ increases the PLDα1 enzyme activity without increasing the protein production because the cellular concentration of PLDα1 remains the same [123].

The PLDα1-PA system stimulates NADPH oxidase activity, resulting in ROS production [127]. This is in agreement with the observation in Arabidopsis, where ROS production increased upon PA treatment [128].

**Figure 4:**
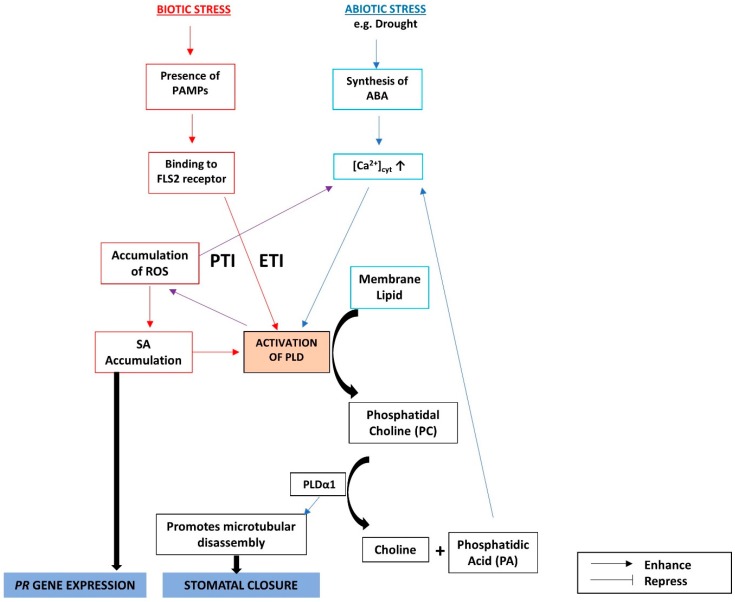
The crosstalks among ABA, SA, and phospholipid signaling during biotic and abiotic stress responses act through PLDs. PTI: PAMP-triggered immunity; ETI: effector-triggered immunity.

During biotic stress, pathogen-associated molecular patterns (PAMPs) such as flg22 are recognized by the cell surface receptors of plants, initiating PAMP-triggered immunity (PTI). To circumvent this plant defense, bacteria and fungi produce effector proteins that disrupt PTI. In turn, plants produce intracellular resistance (R) proteins that can bind to pathogen-produced effector molecules and initiate effector-triggered immunity (ETI) [129]. Hence, a model is proposed, where upon pathogen infection, PAMPs activate PLD-mediated SA accumulation by the PA–ROS–SA pathway in two waves. The first peak of SA accumulation is rapid (0.5 to 2 h) during PTI and/or ETI. Accumulated SA activates PLD, which leads to a second wave of SA accumulation (after 2 to 10 h) by the PA–ROS–SA pathway, during ETI [130]. The crosstalks among SA, ABA, and phospholipid signaling are summarized in Figure 4.

## 4. Roles of G-proteins in Biotic and Abiotic Stress Responses

G-proteins are cellular molecules for relaying signals. The involvement of G-proteins in JA signaling has been discussed in Section 3 (Figure 1). Upon binding to GTP, G-proteins trigger the hydrolysis of GTP to GDP, and their conformations are changed to the active forms. The active G-proteins bind to downstream targets to modulate their functions. Many G-proteins are stress-responsive. Therefore, they have been regarded as important players in regulating signaling events upon stresses. G-proteins can be categorized into heterotrimeric G-proteins, unconventional G-proteins, and small G-proteins. Some G-proteins, such as the unconventional G-protein, YchF, have been reported to play dual roles in abiotic and biotic stresses.

### 4.1. Heterotrimeric G-proteins

A heterotrimeric G-protein typically comprises three subunits (α, β, and γ). In plants, a heterotrimeric G-protein consists of one α-subunit (AGA1), one β-subunit (AGB1), and three γ-subunits (AGG1, AGG2, and AGG3). It transduces signals from a G-protein-coupled receptor (GPCR) in response to environmental stimuli.

The Arabidopsis Gα subunit, namely GPA1, plays an important role in stomatal defense against bacterial pathogens. For example, growth of the coronatine-deficient *P. syringae* pv. *tomato* DC3118 is significantly increased in *gpa1* mutant plants [131]. In addition, the rice *d1* mutant, which is deficient in the Gα subunit, exhibits more severe disease symptoms when inoculated with *Xanthomonas oryzae* pv. o*ryzae*, and it has significantly lowered production of the probenazole-inducible protein, PBZ1, which belongs to PR10 proteins and exhibits RNase activity, and thus induces cell death against necrotrophic pathogens, compared to the wild type [132].

On the other hand, using the *bir1-1* mutant of Arabidopsis, the G-protein β-subunit (AGB1) was found to function downstream of the leucine-rich repeat receptor-like kinase (LRRR-RLK), SOBIR1, to initiate cell death [131]. In *agb1* null-mutant plants, the induction of PAMP-triggered defense responses as mediated by three other RLKs: FLS2, EFR, and CERK1, are severely compromised, suggesting that AGB1 is a signaling component for plant immunity in common among pathways mediated by different RLKs [131].

Arabidopsis AGB1 also plays a role in the defense against the necrotrophic pathogens, *Fusarium oxysporum* f. sp. *conglutinans* and *Alternaria brassicicola* (isolate UQ4273). Using the *agb1* mutant against these pathogens, AGB1-mediated signaling was found to suppress the induction of *PR* genes by SA, JA, ethylene, or ABA during the initial phase of the infection. However, with the use of double mutants, leaf necrosis resulting from the *agb1-2* mutation was shown to be rescued by simultaneous *coi1-21* and *jin1-9* mutations. Thus, AGB1 works upstream of COI1 and ATMYC2 in JA signaling during the late phase of infection by necrotrophic pathogens [133].

For the Gγ subunits, AGG1 and AGG2 have redundant roles in Arabidopsis in response to elf18-induced oxidative bursts (a PTI response), although AGG1 seems to play the leading role. However, AGG3 is found to be involved in stomatal ion channel regulation and the development of reproductive organs, and it has no effect on the resistance against necrotrophic pathogens. Thus, AGG3 is probably not involved in PTI [131].

Heterotrimeric G-proteins also participate in abiotic stress responses. The Gα subunit from carrot seedlings were shown to exhibit lowered expression under heat stress and prolonged salt treatment [134,135] Meanwhile Gβ is found to be involved in salt stress tolerance only. Arabidopsis *agb1-2* mutant exhibited lower cotyledon greening rates, fresh weight, root length, seedling germination rates, and survival rates [135].

### 4.2. Unconventional G-proteins

The unconventional G-proteins are unimolecular and are similar in size to the heterotrimeric Gα subunit. They are classified into extra-large G-protein, GPCR-type G-proteins (GTGs), and the Obg superfamily.

In the case of extra-large G-proteins, the Arabidopsis genome contains three genes encoding proteins with limited homology to Gα subunits, but more than twice the size of GPA1, and thus they were named extra-large G-proteins (XLGs) upon their discovery [136]. XLGs contain two distinct domains, an N-terminal cysteine-rich region and a C-terminal Gα-like domain [136]. The xlg mutants share some similar phenotypes with *agb1* mutants, suggesting the existence of functional similarities between XLGs and Gβ. Similar to the *agb1* mutants, *xlg2* mutants displayed increased susceptibility to *Pseudomonas syringae* pv. tomato DC3000. XLG2 interacts directly with AGB1 and acts as a positive regulator in the salicylic acid pathway [137]

Another class of unconventional G-proteins, GPCR-type G-proteins (GTGs), has also been functionally characterized in Arabidopsis [138]. GTGs are multi-unit transmembrane G-proteins with a span topology reminiscent of G-protein-coupled receptors (GPCRs), but with nine predicted transmembrane spans instead of the seven that is characteristic of GPCRs. The Arabidopsis genome encodes two GTG proteins, GTG1 (At1g64990) and GTG2 (At4g27630), which have 90% sequence identity at the amino acid level. Both GTG1 and GTG2 interact with the Arabidopsis GPA1. The *gtg1gtg2* double mutant is defective in a variety of classic ABA responses, including inhibition of seed germination, prevention of cotyledon greening, retardation of primary root elongation, induction of ABA-responsive genes such as *RAB18*, *RD29B*, *DREB2A*, *DREB2B*, and *ERD10*, and promotion of stomatal closure.

#### 4.2.1. Obg Superfamily

The Obg superfamily is a TRAFAC class of P-loop GTPases that have a highly conserved amino acid sequence in various organisms from prokaryotes to eukaryotes, and are thus believed to be the descendants of ancient proteins. Some of them are found to play important roles in basic cellular processes, e.g., ribosome biogenesis, translation, and signal transduction. This superfamily includes the Obg, EngB, HflX, Era, TrmE, EngA, EngD/ YchF, Drg, and Nog1 G-protein families. 

##### Obg/Era Family

Spo0B-associated GTP-binding (Obg) proteins was named as such because it was found to be downstream to the *Spo0B* gene in *Bacillus subtilis*. It is found to be essential for the viability of nearly all bacteria. However, its functions in plant are least to be studied. Rice *OsTSV3* encodes a Spo0B-associated GTP-binding (Obg) protein was reported to exhibit functions related to cold stress responses. The cold-sensitive virescent *tsv3* mutant of rice exhibits an albino phenotype at 20 °C, but develops normal green leaves at 32 °C before the three-leaf stage, and has a normal green phenotype at 20 °C after the 4-leaf stage. The expressions of genes associated with the biogenesis of the chloroplast ribosome 50S subunit in the *tsv3* mutant were severely decreased in the three-leaf stage seedlings at 20 °C. This observation implied that the nuclear-encoded Obg G-protein member, TSV3, plays critical roles in chloroplast development at early developmental stages [139].

##### Drg Family

Developmentally regulated GTP-binding proteins (DRGs), being one of the family in the Obg superfamily, belongs to the Archaea-related type with Nog1. As suggested by the name, its functions are mainly related to development. However, current findings showed that it is also related to cold stress responses. Arabidopsis *AtDRG1-3* is induced during seed drying and heat stress [140]. The expressed protein of AtDRG1-3 is localized not only in the cytoplasm, such as AtDRG1-1 and AtDRG1-2, but also in the nucleus. However, the function of *AtDRG1-3*, which is stress inducible, is not yet elucidated [140].

##### YchF Family

The YchFs are a group of ancient proteins, and their sequences are highly conserved among all kingdoms of life except Archaea [141]. The rice YchF1 protein (OsYchF1) was the first plant YchF protein, with its physiological functions characterized in relation to both biotic and abiotic stresses [141,142].

OsYchF1 is the interacting partner of a wounding-inducible protein, *Oryza sativa* GTPase-activating protein 1 (OsGAP1). OsGAP1 is a protein specific to higher plants, and it is induced during wounding in the bacterial blight-resistant rice line that expresses the *R* gene, *Xa14*, against bacterial blight. OsYchF1 is identified as its protein interacting partner through yeast two-hybrid experiments, in vitro pull-down and bimolecular fluorescence complementation (BiFC). OsGAP1 not only interacts with OsYchF1, but it also regulates OsYchF1 in several aspects. OsGAP1 alters the subcellular localization of OsYchF1, its RNA-binding affinity, and the substrate specificity of NTP hydrolysis [141].

More importantly, OsGAP1 is able to enhance plant resistance towards bacterial pathogens. OsGAP1-transgenic rice and Arabidopsis exhibit phenotypes with fewer disease lesions after inoculations with *Xanthomonas oryzae* pv. *oryzae* (*Xoo*) and *Pseudomonas syringae* pv. *tomato* DC3000 (*Pto* DC3000), respectively [143]. Besides having less severe disease symptoms, the OsGAP1-expressing transgenic plants also have lower pathogen titers from infected leaves, and higher induction of *PR* genes involved in the SA, JA, and ethylene pathways [143]. In addition, the ectopic expression of OsGAP1 in the Arabidopsis *npr1-3* mutant failed to enhance resistance against *Pto* DC3000, and could not induce the above-mentioned *PR* genes, thus demonstrating that the function of OsGAP1 on biotic stress is upstream of NPR1, which is a well-known key regulator in biotic stress responses, and plays important roles as the convergence point of several signaling pathways, including the SA-dependent systemic acquired resistance, as well as the JA/ET-dependent induced systemic resistance [143].

However, the positive role of OsGAP1 in biotic stress responses function through its deactivation of OsYchF. Arabidopsis transgenic lines ectopically expressing OsYchF1 show increased susceptibility to *Pto* DC3000 [141]. Thus, it is speculated that OsGAP1 acts as a positive regulator in disease resistance pathways by stimulating the GTPase/ATPase activities of OsYchF1, and thus converting it into the GDP-/ADP-bound inactive form. Meanwhile, OsGAP1 also localizes OsYchF1 from the cytosol to the plasma membrane upon wounding. In addition, OsGAP1 interacts with OsYchF1 at its TGS domain, which is a ribonucleic acid-binding domain. OsYchF1 is able to bind the rice 26S rRNA, and OsGAP1 was found to compete with 26S rRNA for binding to OsYchF1 via in vitro pull-down experiments. Therefore, the function of OsYchF1 as a negative regulator for biotic stress is suppressed by OsGAP1 [141].

YchF1 and GAP1 proteins not only play roles in biotic stress, they are also involved in abiotic stress responses. The ectopic or over-expression of rice and Arabidopsis YchF1 proteins in transgenic Arabidopsis enhances the sensitivity of the plants towards salt stress. On the other hand, *AtYchF1*-knockdown mutant exhibits higher tolerance towards salt treatment when compared to the wild type. Meanwhile, transgenic Arabidopsis lines over-expressing *AtGAP1* demonstrated higher salt tolerance when compared to the wild type while *AtGAP1*-knockdown lines showed lower salt tolerance [142]. Higher anti-oxidant activities were also found in the *AtYchF1*-knockdown mutant, *AtGAP1*-over-expressing lines, and *OsGAP1*-transgenic Arabidopsis lines. In addition, *OsYchF1*-transgenic tobacco BY-2 cells show higher levels of ROS generation and percentage of cell death under salt treatment [142].

Therefore, YchF1 acts as a negative regulator to suppress both biotic and abiotic stress responses under favorable growing conditions. When there are stresses present, *GAP1* is induced, and the GAP1 protein would inactivate YchF1 and then trigger defense responses.

One of the special features of YchF1 is its lower nucleotide-binding specificity than typical GTPases; it can bind both GTP and ATP. By resolving the structure of OsYchF1 via crystallization, it is deduced that the G4 motif of OsYchF1 is responsible for the lowered specificity, and therefore its lower ability to use both ATP and GTP as a substrate. The canonical sequence of the G4 motif for GTP specificity is NKXD, while the sequence of the OsYchF1 G4 motif is NMSE. Mutation of the native noncanonical sequence of the G4 motif of OsYchF1 to the canonical sequence (i.e., mutating NMSE to NKSD) precludes the binding/hydrolysis of ATP by OsYchF1. Transgenic Arabidopsis over-expressing the mutated versions of OsYchF1-G4 and AtYchF1-G4 could only bind GTP but not ATP, and could no longer function as a negative regulator of plant defense responses. On the other hand, being only able to bind/hydrolyze GTP and not ATP still allows YchF1 to function as a negative regulator of abiotic stress responses. This study showcases the specific nature of the ATP-binding/hydrolyzing ability of YchF1 in determining its function in disease resistance [144].

### 4.3. Small G-proteins

In animals, there are two subfamilies within the Ras superfamily of small G-proteins: Ras and Rho. However, plants do not have Ras, but they contain Rho-like small G-proteins called RACs or ROPs.

The Arabidopsis genome encodes a family of 11 ROP small GTPases, while the rice genome comprises seven members and there are six *ROPs* in the barley genome [145,146].

The rice Rac protein (OsRAC1) is involved in defense against *Magnaporthe grisea* and elicitor (sphingolipid)-triggered responses. It positively regulates NADPH oxidase and cinnamoyl-CoA reductase, so as to increase ROS production and lignin biosynthesis, respectively [147].

Overexpression of a Ras-related small G-protein, RGPL, in *Nicotiana tabacum* results in higher production of SA and pathogenesis-related (PR) proteins, and increases resistance towards tobacco mosaic virus (TMV) [148].

In response to abiotic stress, Arabidopsis Rac1 (AtRac1) responds to ABA by disrupting the actin skeleton in guard cells, and this eventually leads to stomatal closure during drought conditions [149].

Transgenic rice and Arabidopsis lines overexpressing *OsRAN2* exhibit increased sensitivity towards salt (NaCl) and osmotic stress (PEG6000), as well as exogenous ABA (10 μM). Meanwhile, transgenic rice lines with knocked-down *OsRAN2* expressions showed the reverse phenotypes. *OsRAN2* gene expression declines to around 20% to 40% of that in the wild type during the first 10 h of treatments, including salt stress, osmotic stress, and ABA treatment. Besides, both rice and Arabidopsis transgenic lines with higher or ectopic *OsRAN2* expressions develop shorter and fewer roots, and smaller leaves under the aforementioned stress conditions. To conclude, the OsRAN2 protein is a negative regulator in abiotic stress responses [150].

On the other hand, OsRAN2 plays a positive role under cold stress. *OsRAN2* was induced during cold stress, and transgenic lines over-expressing *OsRAN2* showed higher survival rates under cold treatment, where over 50% of seedlings were able to grow and stay green after a two-week recovery period in normal greenhouse conditions when compared to the wild type, where only around 20% survived after the recovery period. OsRAN2 was found to maintain cell division via promoting the export of intra-nuclear tubulin at the end of mitosis, and it was thus able to maintain the normal nuclear envelope under cold stress [151].

A list of the different classes of G-proteins and their roles in abiotic and biotic stress responses is presented in Table 3.

## 5. Conclusions

Upon environmental challenges, a huge network of signaling events occur in the plant cell. Ca^2+^ sensors are responsible for decoding the natures of the stimuli, and the signals are then transduced through the appropriate stress hormone pathways to bring forth a series of physiological responses. Some major plant hormones, such as ABA and JA, are closely coordinated with Ca^2+^ signaling. Therefore, it has been speculated that ABA and JA are the convergent points between abiotic stresses and biotic stresses. This speculation is supported by studies showing the dual roles of Ca^2+^ sensors as both the initial stress signal detectors and also the regulators of ABA and JA signaling. At the same time, G-proteins are also known to play dual roles in abiotic stress and biotic stress responses, and it has been reported that G-proteins are also involved in the signaling pathways of plant hormones, such as JA. However, as G-proteins consist of so many different sub-groups (Table 3), their diverse behaviors could be an obstacle to fully understanding the roles of G-proteins in plant hormone signaling events. Nevertheless, the diverse natures of G-proteins also hint at the many possibilities of how G-proteins can regulate plant hormone signaling under biotic and abiotic stresses.

## Figures and Tables

**Table 1:** Stress-responsive Ca^2+^ sensors in plants.

Ca^2+^ Sensor	Plant Species	Gene	Stress	Response	Treatment Description	Positive/Negative Regulator	Reference
Calmodulin (CaM)	*Solanum lycopersicum*	*SlCaM1* to *SlCaM6*	Mechanical wounding, *Botrytis cinerea* infection	The expressions of all six *SlCaM* genes were induced by mechanical wounding and *Botrytis cinerea* infection. Transgenic tomato overexpressing *SlCaM2* was more resistant to *Botrytis cinerea* infection.	Mechanical wounding: the tomato fruit pericarp was manually cut into one inch-pieces using a sharp knife. *Botrytis cinerea* infection: mechanically injured tomato fruit was inoculated with *Botrytis cinerea* strain 22B conidial suspension.	*SlCaM2* was a positive regulator. The other five genes were not tested.	[15]
	*Arabidopsis thaliana*	*AtCaM3*	Heat	*Arabidopsis thaliana* overexpressing *AtCaM3* had improved tolerance to heat shock; *cam3* mutants were less tolerant to heat shock.	Six-day-old seedlings on phytagel plates supplemented with Murashige and Skoog medium and sucrose were exposed to 45°C for 50 min or 70 min before the recovery at 22°C for six days.	Positive regulator of heat stress.	[16]
	Soybean	*SCaM-4*	Non-specific fungal elicitor prepared from *Fuasrium solani*, *Phytophthora parasitica* pv. *nicotianae* infection.	The expression of *SCaM-4* was induced by a non-specific fungal elicitor prepared from *Fuasrium solani* to soybean suspension cell culture (SB-P). Overexpression of *SCaM-4* in *Nicotiana tabacum* conferred enhanced resistance to *Phytophthora parasitica* pv. *nicotianae* infection.	Soybean suspension cell culture (SB-P) was treated with a non-specific fungal elicitor prepared from *Fuasrium solani*. Transgenic *Nicotiana tabacum* overexpressing *SCaM-4* was inoculated with *Phytophthora parasitica* pv. *nicotianae* by syringe infiltration into leaves.	Positive regulator of *Fuasrium solani*, *Phytophthora parasitica* pv. *nicotianae* infection.	[17]
		*SCaM-5*	Non-specific fungal elicitor prepared from *Fuasrium solani*, *Phytophthora parasitica* pv. *nicotianae* infection.	The expression of *SCaM-5* was induced by a non-specific fungal elicitor prepared from *Fuasrium solani* to soybean suspension cell culture (SB-P). Overexpression of *SCaM-4* in *Nicotiana tabacum* conferred enhanced resistance to *Phytophthora parasitica* pv. *nicotianae* infection.	Soybean suspension cell culture (SB-P) was treated with a non-specific fungal elicitor prepared from *Fuasrium solani*. Transgenic *Nicotiana tabacum* overexpressing *SCaM-5* was inoculated with *Phytophthora parasitica* pv. *nicotianae* by syringe infiltration into leaves.	Positive regulator of *Fuasrium solani*, *Phytophthora parasitica* pv. *nicotianae* infection.	[17]
Calmodulin-like protein (CML)	*Arabidopsis thaliana*	*AtCML9*	Salt, cold, dehydration, ABA treatment	The expression of *AtCML9* was induced by NaCl, cold, and ABA treatments. The expression of *AtCML9* was induced by dehydration in the first 10 min, but the expression level decreased from 10 min to 40 min after the treatment. The *cml9* mutant had increased sensitivity to ABA and enhanced tolerance to salt and dehydration.	Salt: 10-day-old *Arabidopsis thaliana* seedlings were transferred to agar plates supplemented with 150 mM NaCl for expression study. *Arabidopsis thaliana* seeds were sown onto filter paper saturated with 200mM NaCl or 400mM mannitol before imbibition and germination assay. Three-week-old *Arabidopsis thaliana* was grown on soil and irrigated with 150 mM NaCl every three days, with the phenotype monitored for two weeks. Cold: 10-day old *Arabidopsis thaliana* seedlings on agar plates were exposed at 4°C under light. ABA: 10-day-old *Arabidopsis thaliana* seedlings were sprayed with 100 µM ABA for expression study. *Arabidopsis thaliana* seeds were sown on Murashige and Skoog (MS) medium supplemented with 0.2 µM ABA for cotyledon opening and greening assay. Dehydration: excised leaves of *Arabidopsis thaliana* were desiccated in growth chamber for expression study. Watered 3-week-old *Arabidopsis thaliana* grown on soil had the irrigation withheld for 11 days before re-watering.	Negative regulator of salt stress and dehydration	[19]
			*Pseudomonas syringae* pv. *tomato* (*Pto*) strain DC3000 infection	The expression of *AtCML9* was induced by *P. syringae* pv. *tomato* (*Pto*) strain DC3000 infection half an hour and one hour after infection, but repressed three hours after the infection. The expression response of *AtCML9* to flagellin application was similar to that after *Pto* strain DC3000 infection. *Arabidopsis thaliana* overexpressing *AtCLM9* was more resistant to *Pto* strain DC3000 infection, while *cml9* mutant was more sensitive to *the* infection.	Flagellin application: Arabidopsis thaliana seedlings were grown for 11 days on MS medium. 1 µM flg22 was applied to the fresh MS medium on the ninth day for gene expression study. *P. syringae* pv. *tomato* (*Pto*) strain DC3000 infection: 4-week-old Arabidopsis thaliana was inoculated with *Pto* strain DC3000 in suspension culture by syringe infiltration on the abaxial side of the leaves.	Positive regulator of *P. syringae* pv. *tomato* (*Pto*) strain DC3000 infection.	[20]
	*Arabidopsis thaliana*	*AtCML37*	Drought	*cml37* mutant was highly susceptible to drought stress.	Four-week-old *Arabidopsis thaliana* were not watered for one week, then re-watered for one week before being not watered for another one week.	Positive regulator of drought stress	[21]
			Herbivory	*cml37* mutant was more susceptible to herbivory. CML37 deregulates the JA pathway.	Five-week-old *Arabidopsis thaliana* were subject to feeding by *Spodoptera littoralis* larvae for 24 or 48 h.	Positive regulator of herbivory	[22]
		*AtCML42*	Herbivory, UV-B, drought	*cml42* mutant was more resistant to herbivory but less tolerant to UV-B. *cml42* mutants had higher levels of ABA under drought stress.	Herbivory: Five-week-old *Arabidopsis thaliana* was subjected to feeding by *Spodoptera littoralis* larvae for 24 h. UV-B: *Arabidopsis thaliana* grown on MS plates for eight days were exposed to UV-B for one hour at the intensity of 100 μW∙cm^−2^ and then allowed to grow for five weeks. Drought: Three-week-old *Arabidopsis thaliana* were unwatered for 16 days for survival study, and were unwatered for eight days, rewatered, and then unwatered for eight days for measuring ABA level.	Positive regulator of UV-B stress, negative regulator of herbivory. The drought-resistant phenotype was uncertain.	[23]
	*Solanum habrochaites*	*ShCML44*	Cold, drought, osmotic stress, salt, ABA and JA treatments	The expression of *ShCML44* was induced by cold, drought, osmotic stress, salt, and ABA treatments. Transgenic tomato plants overexpressing *ShCML44* were more tolerant to cold, drought, and salinity stresses.	Six-week-old seedlings were put into a growth chamber for five days as an adaptation period before treatment. Cold: seedlings were transferred to a growth chamber at 4 °C. Drought: seedlings were uprooted, washed, and dehydrated on filter paper. Salt: seedlings were irrigated with 200 mM NaCl. ABA treatment: seedlings were sprayed with 100 µM ABA. JA: seedlings were sprayed with 100 µM MeJA.	Positive regulator of cold, drought, and salinity stresses.	[18]
Calcineurin-B-like protein (CBL)	*Arabidopsis thaliana*	*AtCBL5*	Drought, salt	Overexpression of *AtCBL5* in *Arabidopsis thaliana* improved tolerance to drought and salt stresses.	Drought: 4-week-old *Arabidopsis thaliana* grown on potting soil were unwatered for three weeks. Salt stress: 4-week-old *Arabidopsis thaliana* grown on potting soil were treated with 300 mM NaCl once every three days for two weeks.	Positive regulator of drought and salt stresses.	[26]
	*Brassica napus*	*BnCBL1*	Salt stress, osmotic stress, low inorganic phosphate (Pi), ABA treatment.	The expression of *BnCBL1* was induced by salt stress, osmotic stress, low Pi, and ABA treatment. Overexpression of *BnCBL1* conferred improved tolerance to salt stress and low Pi.	Salt stress: 1-week-old seedlings of *Brassica napus* were transferred to MS medium containing 150 mM NaCl for expression study. Transgenic *Arabidopsis thaliana* seedlings were transferred to MS medium containing 0 to 250 mM NaCl for stress tolerance study. Osmotic stress: 1-week-old seedlings of *Brassica napus* were transferred to MS medium containing 200 mM mannitol for expression study. ABA treatment: 1-week-old seedlings of *Brassica napus* were transferred to MS medium containing 100 μM ABA for expression study. Low Pi: 1-week-old seedlings of *Brassica napus* were transferred to MS medium containing 10 μM phosphate for expression study. Six-day-old transgenic *Arabidopsis thaliana* seedlings were transferred to 50 μM low phosphate (LP) medium for a few days.	Positive regulator of salt stress and low Pi.	[25]
	*Arabidopsis thaliana*	*AtCBL10*	Salt	*cbl10* mutant was more sensitive to salt stress.	Four-week-old *Arabidopsis thaliana* plants were treated with 300 mM NaCl once every three days for two weeks.	Positive regulator of salt stress.	[27]
	*Solanum lycopersicum*	*SlCBL10*	*Pseudomonas syringae* pv *tomato* (*Pto*) strain DC3000 infection	Silencing of *SlCBL10* led to improved resistance to *Pto* strain DC3000 infection.	*Solanum lycopersicum* plants were infected with *Pto* strain DC3000.	Negative regulator of *Pto* strain DC3000 infection.	[28]
Calcium-dependent protein kinase (CPK)	*Arabidopsis thaliana*	*AtCPK10*	Drought	*cpk10* mutant plants were more sensitive to drought stress. Overexpression of *AtCPK10* conferred improved tolerance to drought stress.	One-week-old seedlings were grown for 20 days with or without watering.	Positive regulator of drought stress.	[32]
		*AtCPK6*	PEG-induced drought stress, salt	Overexpression of *AtCPK6* in *Arabidopsis thaliana* conferred tolerance to drought and salt stress.	Drought stress: 3-week-old plants grown in potting soil were watered with 15% polyethylene glycol (PEG) for two weeks. Salt stress: 3-week-old plants grown in potting soil were watered with 250 mM NaCl for two weeks.	Positive regulator of drought stress and salt stress.	[34]
	*Zea mays*	*ZmCPK4*	Drought, ABA	Overexpression of *ZmCPK4* in *Arabidopsis thaliana* conferred tolerance to drought stress and increased sensitivity to ABA.	ABA treatment: *Arabidopsis thaliana* seeds were planted on MS medium supplemented with 0, 1, 2, or 5 µM ABA for germination study. Four-day-old *Arabidopsis thaliana* seedlings were transferred to MS medium with 50 µM ABA for phenotypic study. Rosette leaves of *Arabidopsis thaliana* were treated with 10 µM ABA under light for two hours for stomatal aperture study. Drought: 4-week-old *Arabidopsis thaliana* plants were subject to drought stress by withholding water for 25 days.	Positive regulator of drought stress and ABA sensitivity.	[36]
	*Oryza sativa*	*OsCPK9*	Drought, ABA, salt, osmotic stress.	The expression of *OsCPK9* was induced by ABA, NaCl and osmotic stress. Overexpression of *OsCPK9* in *Oryza sativa* conferred increased tolerance to drought stress. Silencing of *OsCPK9* led to reduced tolerance to drought stress.	ABA: 2-week-old *Oryza sativa* seedlings were transferred to plastic boxes containing 100 μM ABA for 24 h for expression study. Salt: 2-week-old *Oryza sativa* seedlings were transferred to plastic boxes containing 200 mM NaCl for 24 h for expression study. Osmotic stress: 2-week-old *Oryza sativa* seedlings were transferred to plastic boxes containing 20% PEG-6000 for 24 h for expression study. Drought: 3-week-old *Oryza sativa* seedlings were deprived of water for 20 or 27 days before recovery with watering for three days for tolerance study.	Positive regulator of drought stress and ABA sensitivity.	[37]
		*OsCPK12*	Salt, *Magnaporthe grisea* infection	Overexpression of *OsCPK12* in *Oryza sativa* conferred increased tolerance to salt stress and increased sensitivity to ABA. Silencing and mutation of *OsCPK12* led to increased sensitivity to salt stress. Overexpression of *OsCPK12* in *Oryza sativa* conferred increased sensitivity to blast fungus, Ina86-137.	Salt stress: 2-week-old seedlings were exposed to 200 mM NaCl solution for five days. ABA treatment: 5-day-old seedlings were transferred to Yoshida’s nutrient solution supplemented with 0.5 µM ABA for two weeks. *Magnaporthe grisea* infection: agar slice with *Magnaporthe grisea* was attached to wounded leaves of 2–4-week-old *Oryza sativa* seedlings.	Positive regulator of salt stress, negative regulator of *M. grisea* infection.	[38]
Calcium/calmodulin-dependent protein kinase (CCaMK)	*Glycine soja*	*GsCBRLK*	Cold, ABA, salt, osmotic stress	The expression of *GsCBRLK* was induced in leaf by cold, ABA, NaCl, and PEG treatments. The expression of *GsCBRLK in root had diverse responses to* cold, ABA, NaCl and PEG. Overexpression of *GsCBRLK* in *Arabidopsis thaliana* led to improved tolerance to NaCl and reduced sensitivity to ABA.	Cold: 1-month-old soybean seedlings were incubated at 4°C for 0.5, 1, 3, or 6 h for expression study. ABA treatment: 1-month-old soybean seedlings were treated with 100 µM ABA for 0.5, 1, 3, or 6 h for expression study. Twenty-one-day-old transgenic *Arabidopsis thaliana* plants were treated with 100 µM ABA for stress response study. Salt: 1-month-old soybean seedlings were treated with 200 mM NaCl for 0.5, 1, 3, or 6 h for expression study. 21-day-old transgenic *Arabidopsis thaliana* plants were treated with 200 mM NaCl for stress response study. Osmotic stress: 1-month-old soybean seedlings were treated with 30% PEG 6000 for 0.5, 1, 3, or 6 h for expression study.	Positive regulator of salt stress but negative regulator of ABA sensitivity	[29]
	*Solanum lycopersicum*	*SlCCaMK*	*Sclerotinia sclerotiorum* infection, *Pseudomonas syringae* pv. *tomato* (*Pto*) DC3000 infection, *Xanthomonas oryzae* pv. *oryzae* (*Xoo*) infection	The expression of *SlCCaMK* was induced in leaf by *S. sclerotiorum* infection but repressed in leaf by *Pto* DC3000 /*Xoo* infection. Knock-down of *SlCCaMK* led to reduced resistance to *S. sclerotiorum* and *Pto* DC3000 infections.	*Sclerotinia sclerotiorum* infection: *S. sclerotiorum* was inoculated into leaves of 7–8-week-old *Solanum lycopersicum* for expression study. *Pto* DC3000 infection: bacterial suspension culture was infiltrated into leaves for stress response study. *Xoo* infection: bacterial suspension was infiltrated into leaves for stress response study.	Positive regulator of *S. sclerotiorum* and *Pto* DC3000 infections.	[40]
	*Triticum aestivum*	*TaCCaMK*	Salt, PEG-induced drought stress, ABA treatment	The expression of *TaCCaMK* was reduced by NaCl, PEG, and ABA treatments. Overexpression of *TaCCaMK* in *Arabidopsis thaliana* led to decreased sensitivity to ABA and improved tolerance to NaCl.	Salt: 7-day-old *Triticum aestivum* seedlings were treated with 200 mM NaCl in Hoagland’s solution for expression study. Seeds of transgenic *Arabidopsis thaliana* was germinated on MS agar supplemented with 0, 50, 100, 150 or 200 mM NaCl for a germination assay. Four-day-old seedlings of transgenic *Arabidopsis thaliana* grown on MS agar were transferred to MS agar supplemented with 0, 100, 170, or 200 mM NaCl for 10 days for phenotypic study. PEG-induced drought stress: 7-day-old *Triticum aestivum* seedlings were treated with 16% PEG in Hoagland solution for expression study. ABA treatment: *7-*day-old *Triticum aestivum* seedlings were treated with 5 µM ABA in Hoagland’s solution for expression study. Seeds of transgenic *Arabidopsis thaliana* were germinated on MS agar supplemented with 0, 1, or 3 µM ABA for germination assay. Four-day-old seedlings of transgenic *Arabidopsis thaliana* grown on MS agar were transferred to MS agar supplemented with 0, 5, 10, 20, 40, or 80 µM ABA for 10 days for phenotypic study.	Positive regulator of salt stress, negative regulator of ABA sensitivity.	[43]

**Table 2:** Stress-responsive Ethylene Response Factors (ERFs).

Plant	ERF	Induction by	Target Gene Promoter Sequence	Results of Overexpression	References
Pepper	CaPF1	*Xanthomonas axonopodis*	GCC box/DRE sequence	Resistance to disease and cold	[88]
Cotton	GhERF6	Ethylene, ABA, salt, cold, and drought	GCC box	Resistance to salt, cold & drought	[90]
Soybean	GmERF3	Ethylene, ABA, SA, JA, Soybean Mosaic Virus, dehydration, salt	GCC box/DRE sequence	Resistance to disease, drought and high salt, induction of *PR* genes	[78]
Tomato	JERF1	Ethylene, MeJA, ABA, and salt	GCC box/DRE sequence	Resistance to salt and cold, induction of the ABA biosynthesis-related gene *NtSDR*, accumulation of ABA	[81]
	JERF3	Ethylene, JA, ABA, cold, salt	GCC box/DRE sequence	Resistance to salt, induction of *PR* genes	[77]
	LeERF3b (class II)	Ethylene, cold, drought	GCC box/DRE sequence/C-repeat	Cold tolerance	[86]
	SlERF5	High salinity, drought, flooding, wounding and cold	GCC box	Resistance to drought and salt	[80]
Wheat	TaERF3	Salt, polyethylene glycol (PEG)	GCC box	Resistance to drought and salt	[79]
	TaERF7	Drought, salt, MeJA, ethylene and ABA. Depressed by cold	GCC box	Resistance to salt, accumulation of soluble carbohydrates and decreased concentration of malondialdehyde, susceptibility to cold	[89]
	TaPIE1	Ethylene, *Rhizoctonia cerealis* and freezing stresses	GCC box	Resistance to *R. cerealis* and freezing stress, higher accumulation of soluble sugars and proline	[87]
Rice	OsWR1	Drought, ABA and salt	GCC box/DRE sequence	Induction of wax/cutin synthesis genes	[91]
	OsEREBP1	*Xanthomonas oryzae*	GCC box	Resistance to cold, salinity, drought & submergence, induction of genes for JA and ABA biosynthesis, lipid metabolism, alcohol dehydrogenases (related to submergence), and *PR* genes	[75,92]
Tobacco	OPBP1	Cryptogein, salt, ethephon, MeJA, cycloheximide.	GCC box	Resistance to pathogen and salt stress, induction of *PR* genes	[93]
	Tsi1	Salt, ethephon, SA	GCC box/DRE sequence	Resistance to pathogen and salt, induction of *PR* genes	[76]

**Table 3:** G-proteins and their roles in biotic and abiotic stress responses.

Class of G-Protein	Plant Species	Gene	Stress	Response	Treatment Description	Positive/Negative Regulator	Reference
Heterotrimeric G-protein α subunit	*Arabidopsis thaliana*	*AGA1*	*Pseudomonas syringae* pv. *tomato* (*Pto*) DC3000 infection	*aga1* mutant failed to close stomata after coronatine treatment, and thus exhibited higher susceptibility to *Pto* DC3000 infection	Five-week-old seedlings were dipped upside down in coronatine (COR)-deficient mutant *Pto* DC3000 bacterial suspension for a few seconds	Positive regulator of coronatine-induced stomatal closure, and in turn, stomatal defense against *Pto* DC3000 infection.	[152]
	*Oryza sativa*	*D1*	*Xanthomonas oryzae* pv. *oryzae* infection	*d1* mutant, which is deficient in G-protein α subunit failed to defend against *X. oryzae* pv. *oryzae* infection and exhibited delayed induction of probenazole-inducible protein (PBZ1)	At four weeks after sowing, the uppermost fully opened leaves were inoculated by the double-needle pricking and cutting method	Positive regulator of bacterial blight resistance. Acts through the phosphorylation of both the 48-kDa putative MAPK and the 55-kDa putative CDPK, and induces PBZ1 production	[132]
Heterotrimeric G-protein β subunit	*Arabidopsis thaliana*	*AGB1*	*Hyaloperono-spora arabidopsidis* Noco2 and *Pseudomonas syringae* pv. *tomato* (*Pto*) DC3000 infections	*agb1* mutant is identified to suppress cell death and defense response phenotypes of *bir1-1* mutant	*H. arabidopsidis* infection: 2-week-old seedlings were sprayed with spore suspensions of *H. arabidopsidis* Noco2 at a concentration of 50,000 spores per mL water *Pto* DC3000 infection: 5- to 6-week-old seedlings pre-infiltrated with 1 µM flg22, 1 µM elf18, or 200 µg∙mL^−1^ chitin (PAMPs) followed by infiltration with *Pto* DC3000 suspension	Positive regulator of PAMP-trigged responses. Functions downstream of BIR1-1 and plays positive role in salicylic acid (SA) level	[131]
			*Fusarium oxysporum* f. sp. conglutinans and *Alternaria brassicicola* (isolate UQ4273) infections	*agb1* mutant is more susceptible to necrotrophic pathogens (*F. oxysporum* and *A. brassicicola*). At the initial phase of infection, AGB1 works in a pathway independent of SA, JA/ethylene and ABA signalling.	*F. oxysporum* f. sp. conglutinans infection: 2-week-old plants removed from soil were immersed in *F. oxysporum* spore solution (10^6^ spores∙mL^−1^) for 30–60 s, and then replanted in fresh autoclaved soil. Phenotype was recorded by counting the number of yellow-veined leaves. A plant was considered dead when all leaves had turned yellow. *A. brassicicola* infection: 3-week-old Arabidopsis thaliana seedlings were grown in 100% humidity chambers for five days before inoculation with *A. brassicicola* (isolate UQ4273) spore suspension. Number of leaves with spreading lesions was recorded at five days post-inoculation.	Positive regulator against *F. oxysporum* and *A. brassicicola* infections through pathways both dependent on and independent of SA, JA and ethylene.	[133]
Heterotrimeric G-protein γ subunit	*Arabidopsis thaliana*	*AGG1* and *AGG2*	*Pseudomonas syringae* pv. *tomato* (*Pto*) DC3000	*agg1 agg2* double mutant fails to exhibit PAMP-triggered responses	Five- to 6-week-old seedlings were pre-infiltrated with 1 µM flg22, 1 µM elf18, or 200 µg∙mL^−1^ chitin (PAMPs), and then infiltrated with *Pto* DC3000 suspension	Positive regulator of PAMP-triggered responses	[131]
Extra-large G-protein	*Arabidopsis thaliana*	*XLG2*	Infection by virulent and avirulent *Pseudomonas* strains	*xlg2* mutant exhibits higher susceptibility towards both virulent and avirulent *Pseudomonas* strains	Leaves of 5-week-old seedlings were infiltrated with 3 × 10^4^ cfu/mL (for virulent *Pseudomonas syringae* pv. *tomato* DC3000) or 1 × 10^5^ cfu/mL (for avirulent *Pto* avrRpm1 and *P. syringae* pv. *phaseolicola* strains) bacterial suspension	Positive regulator of non-host basal resistance	[137]
Obg protein	*Oryza sativa*	*TSV3*	Cold stress	*tsv3* mutant exhibits albino phenotype under cold stress at early-leaf stages	Seedlings were grown in growth chamber with 12 h light and 12 h dark and at constant temperature of either 20 °C (cold treatment) or 30 °C. Phenotypes were observed at 3- and 4-leaf stages.	Positive regulator of cold stress, associated with biogenesis of chloroplast ribosome 50S subunit at 3-leaf stage under cold stress	[139]
DRG protein	*Arabidopsis thaliana*	*DRG1-3*	Heat	DRG1-3 expression is induced by heat stress after 1-4 h	Arabidopsis wild type seeds were germinated and grown on Jiffy medium under continuous light at 23 °C constant temperature, and heat shock was performed for up to four hours	Positive regulator of heat stress response	[140]
YchF protein	*Oryza sativa* and *Arabidopsis thaliana*	*OsYchF1* and *AtYchF1*	*Xanthomonas oryzae* pv. *oryzae* (*Xoo*) and *Pseudomonas syringae* pv. *tomato* (*Pto*) DC3000 infections	Ectopic expression and over-expression of *OsYchF1* and *AtYchF1* in Arabidopsis enhanced susceptibilities to bacterial infection. Arabidopsis *AtYchF1*- knockdown mutant exhibits enhanced resistance.	Eight-week-old seedlings were inoculated with *Xoo* and *Pto* DC3000 via syringe infiltration on abaxial surface of leaves. Disease lesions, pathogen titers and expressions of PR genes were examined three days after inoculation.	Negative regulator of resistance against *Pto* DC3000	[141]
			Salt stress	Ectopic expression and over-expression of *OsYchF1* and *AtYchF1* in Arabidopsis enhances sensitivity to salt stress. Arabidopsis *AtYchF1*-knockdown mutant is more salt-tolerant	Ten-day-old seedlings grown on MS medium were transferred onto MS medium supplemented with 150 mM NaCl. Chlorosis phenotype, chlorophyll content, lipid peroxidation, and salt-responsive gene expressions were recorded after 10 days of salt treatment	Negative regulator of salt stress	[142]
Small G-protein	Oryza sativa	*OsRAC1*	*Magnaporthe grisea* and *Xanthomonas oryzae* pv. oryzae infections	Over-expression of *OsRac1* shows hypersensitive responses and increases resistance against a virulent race of rice blast fungus (*M. grisea*, race 007) and bacterial blight (*X. oryzae* pv. oryzae, race 1)	*Magnaporthe grisea* inoculation was performed via press-injured spots of 2.0 mm diameter made with a specially designed pressing machine (Fujihara co.) on leaf blades of 60-day-old seedlings. A piece of agar covered with spores was then placed on the injured spots. *Xanthomonas oryzae* pv. oryzae inoculation was done at panicle initiation to bolting stage by clipping off the leaves at 2–3 cm from leaf tip with sterilized scissors and dipping the clipped edge of leaves in the bacterial suspension (approximately 10^9^ cfu/mL).	Positive regulator of resistance to both necrotrophic and biotrophic pathogens	[153]
	*Nicotiana tabacum*	*RGP1*	Tobacco mosaic virus (TMV) infection	Over-expression of a Ras-related small G-protein, RGP1, shows higher production of salicylic acid and PR proteins and increased resistance towards TMV	Wounding was made on fully expanded fifth leaves (leaf 5) by punching out leaf discs from seedlings at 16-leaves stage. For quantitation of SA and SAG, the target leaves were wounded by gentle rubbing of the upper epidermis with wet carborundum. For TMV inoculation, upper fully expanded leaves were detached and inoculated with TMV (10 viral particles /g/mL) by using carborundum (Mesh 600) and incubated at 20 °C under continuous illumination.	Positive regulator of resistance against tobacco mosaic virus infection	[148]
	*Arabidopsis thaliana*	*AtRac1*	Drought and ABA treatments	*rac1* mutant fails to interrupt the ABA-mediated actin skeleton in guard cells during drought condition and thus blocks stomatal closure	10–50 μM ABA was applied to 2-week-old seedlings for 30 min after 48 h induction with 10 μM DEX in white-light condition. Widths and lengths of stomatal opening were measured using a LSM410 inverted confocal microscope.	Positive regulator of stomatal closure during drought condition or ABA treatment	[149]
	*Oryza sativa* and *Arabidopsis thaliana*	*OsRAN2*	Salt, osmotic stress, and ABA treatments	Over-expression of *OsRAN2* in both rice and Arabidopsis lead to shorter and fewer roots and smaller leaves under salt and osmotic stress.	Transgenic Arabidopsis plants with ectopically expressed OsRAN2 were grown on MS agar plates supplemented with 100 mM NaCl, 100 mM KCl. Phenotypes were recorded after two weeks.	Negative regulator of salt and osmotic stress, likely acting through ABA signalling pathway	[150]
			Cold stress	*OsRAN2* expression is induced during cold stress. *OsRAN2*-overexpressing lines show higher survival rates (seedlings being able to grow and stay green) under cold treatment compared to wild type	Transgenic rice lines over-expressing OsRAN2 were germinated in water (as control) or in water containing 100 mM NaCl, 100 mM KCl, 10% PEG 6000, or 10 μM ABA. Phenotypes were observed after five and 10 days. Two-week-old OsRAN2-overexpressing rice lines at the tetraphyllous leaf stage were treated at 4 °C for 72 h. The seedlings were allowed to recover in normal greenhouse conditions for two weeks.	Positive regulator of cold tolerance, by maintaining cell division via promoting the export of intra-nuclear tubulin at the end of mitosis, thus maintaining normal nuclear envelope under cold stress	[151]
